# Dietary Omega-3 Polyunsaturated Fatty-Acid Supplementation Upregulates Protective Cellular Pathways in Patients with Type 2 Diabetes Exhibiting Improvement in Painful Diabetic Neuropathy

**DOI:** 10.3390/nu14040761

**Published:** 2022-02-11

**Authors:** Alfonso M. Durán, W. Lawrence Beeson, Anthony Firek, Zaida Cordero-MacIntyre, Marino De León

**Affiliations:** 1 Center for Health Disparities and Molecular Medicine, Department of Basic Sciences, Loma Linda University School of Medicine, Loma Linda, CA 92350, USA; aduran@llu.edu (A.M.D.); lbeeson@llu.edu (W.L.B.); cordero-macintyre@llu.edu (Z.C.-M.); 2 Center for Nutrition, Healthy Lifestyle and Disease Prevention, School of Public Health, Loma Linda University, Loma Linda, CA 92350, USA; 3 Comparative Effectiveness and Clinical Outcomes Research Center, Riverside University Health System Medical Center, Moreno Valley, CA 92555, USA; a.firek@ruhealth.org

**Keywords:** omega-3, polyunsaturated fatty acids, painful diabetic neuropathy, metabolism, metabolomics

## Abstract

Background: Omega-3 polyunsaturated fatty acids (PUFAs) have been proposed to improve chronic neuroinflammatory diseases in peripheral and central nervous systems. For instance, docosahexaenoic acid (DHA) protects nerve cells from noxious stimuli in vitro and in vivo. Recent reports link PUFA supplementation to improving painful diabetic neuropathy (pDN) symptoms, but cellular mechanisms responsible for this therapeutic effect are not well understood. The objective of this study is to identify distinct cellular pathways elicited by dietary omega-3 PUFA supplementation in patients with type 2 diabetes mellitus (T2DM) affected by pDN. Methods: Forty volunteers diagnosed with type 2 diabetes were enrolled in the “En Balance-PLUS” diabetes education study. The volunteers participated in weekly lifestyle/nutrition education and daily supplementation with 1000 mg DHA and 200 mg eicosapentaenoic acid. The Short-Form McGill Pain Questionnaire validated clinical determination of baseline and post-intervention pain complaints. Laboratory and untargeted metabolomics analyses were conducted using blood plasma collected at baseline and after three months of participation in the dietary regimen. The metabolomics data were analyzed using random forest, hierarchical clustering, ingenuity pathway analysis, and metabolic pathway mapping. Results: The data show that metabolites involved in oxidative stress and glutathione production shifted significantly to a more anti-inflammatory state post supplementation. Example of these metabolites include cystathionine (+90%), S-methylmethionine (+9%), glycine cysteine-glutathione disulfide (+157%) cysteinylglycine (+19%), glutamate (−11%), glycine (+11%), and arginine (+13.4%). In addition, the levels of phospholipids associated with improved membrane fluidity such as linoleoyl-docosahexaenoyl-glycerol (18:2/22:6) (+253%) were significantly increased. Ingenuity pathway analysis suggested several key bio functions associated with omega-3 PUFA supplementation such as formation of reactive oxygen species (*p* = 4.38 × 10^−4^, z-score = −1.96), peroxidation of lipids (*p* = 2.24 × 10^−5^, z-score = −1.944), Ca^2+^ transport (*p* = 1.55 × 10^−4^, z-score = −1.969), excitation of neurons (*p* = 1.07 ×10^−4^, z-score = −1.091), and concentration of glutathione (*p* = 3.06 × 10^−4^, z-score = 1.974). Conclusion: The reduction of pro-inflammatory and oxidative stress pathways following dietary omega-3 PUFA supplementation is consistent with the promising role of these fatty acids in reducing adverse symptoms associated with neuroinflammatory diseases and painful neuropathy.

## 1. Introduction

Painful diabetic neuropathy (pDN) is a common comorbidity of T2DM and has a significant negative impact on patients’ quality of life and productivity. Effective therapeutic interventions are sorely lacking, and there is a great unmet need for novel and safe therapeutics. Currently, treatment does not target the disease process, resulting in only a third of treated patients achieving 50% pain relief, often complicated by side effects [1,2,3]. In part, the development of therapies is complicated because of the inherently variable and complex nature of pDN. Although progress has been made in understanding the pathophysiology of this debilitating condition, attempts to use various targeted therapies have not resulted in either consistent or sustained benefits to patients with pDN. Progress in the development of proper therapies to treat pDN may require a thorough understanding of its pathogenesis to target cellular pathways specifically disrupted by the disease.

Pre-clinical models suggest that the pathophysiology of pDN involves both microvascular and metabolic changes, which ultimately lead to increased oxidative stress, inflammation, and mitochondrial dysfunction [4]. However, corroborating human studies are needed to determine which specific cellular pathways are involved in pDN. In recent years the development of high-throughput metabolomics analysis represents an opportunity to identify critical biomarkers that may serve to elucidate major metabolic and cellular pathways activated during the pathophysiology of different disease states [5,6,7,8,9,10].

An increasing number of studies have implicated omega-3 PUFAs in reducing the adverse effects of chronic neuroinflammatory diseases in both the peripheral and central nervous systems [11,12]. The treatment with docosahexaenoic acid (DHA) has shown significant protection in nerve cells both in cell culture and in vivo [13,14,15,16]. In addition, it has been shown that dietary omega-3 PUFAs protect against spinal cord injury-induced chronic pain [7] and oral administration can stimulate nerve regeneration and protect peripheral nerves from injury [11,17]. Further, omega-3 PUFAs supplementation promotes nerve regeneration in patients with type 1 diabetes [18,19,20].

In a previous study, we found that participants in a diabetic health education program showed a significant improvement in the sensory pain scores after three months of PUFA supplementation, as assessed with the Short-Form McGill Pain Questionnaire (SF-MPQ) [10]. The data also showed that change in DHA plasma levels significantly correlated with the improved pain status. To better understand the significance of these findings, we postulated that further interrogating the metabolome may identify principal cellular pathways associated with this positive effect of omega-3 PUFAs in humans and provide direction towards identifying metabolites with clinical potential. The present study addresses this hypothesis by characterizing the impact of omega-3 PUFA dietary supplementation on the metabolome in participants with T2DM and exhibiting pDN symptoms.

## 2. Materials and Methods

### 2.1. Study Design and Population

**Scheme 1:**
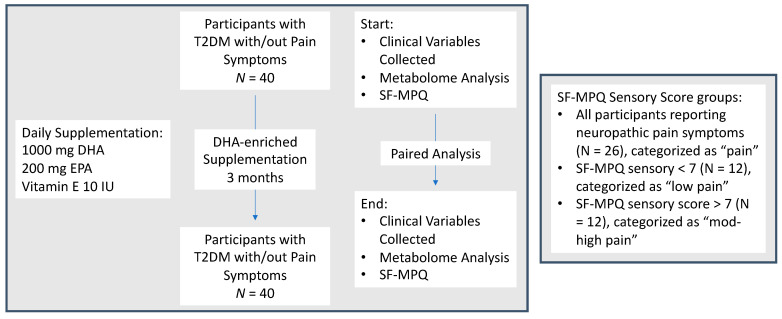
Study Description. DHA, Docosahexaenoic acid; EPA, Eicosapentaenoic acid; T2DM, type 2 diabetes mellitus; SF-MPQ, Short-form McGill Pain Questionnaire.

En Balance-Plus is a longitudinal single-arm designed study conducted in Loma Linda, California, to assess the impact of nutrition and diabetes education on type 2 diabetes among Latinos, primarily Mexican Americans. After three months, the study evaluated the effect of the dietary DHA-enriched supplementation on participants’ metabolomic profiles. The cohort was previously described in detail [10] and a descriptive summary is presented in Scheme 1. After informed written consent was obtained, self-identified Latino Americans with type II diabetes completed a three-month group-based intervention that consisted of consuming dietary DHA-enriched capsules and attending weekly diabetes-education classes conducted in Spanish. Each participant was interviewed in person to obtain diabetes history, medication use, diet, and physical activity habits. The study was approved by the Loma Linda University Institutional Review Board. A total of 40 Latino adults, previously diagnosed with type 2 diabetes between 33 and 74, completed the three-month study. Five participants were excluded from clinical data analysis: one due to a lab reporting error and four due to missing clinical data. The research subjects participated in weekly lifestyle and nutrition classes. They were instructed to consume a daily intake of 1000 mg DHA and 200 mg eicosapentaenoic acid (EPA) (provided by the study) for the duration of the study. In addition to the health-education classes, each participant was contacted weekly by phone to confirm compliance.

Every volunteer in the diabetes-education program completed the SF-MPQ form at the start and at the end of the three months. The SF-MPQ is validated for monitoring patients with pDN and for assessing interventions for pain-improvement outcomes [21]. At baseline, 26 participants reported neuropathic pain symptoms, categorized as the pain group. The data obtained using the SF-MPQ were further analyzed based on the severity of the neuropathic pain symptoms reported. Participants with a sensory score <7 were denominated as the low pain group (*n* = 14) and participants with sensory >7 designated as moderate–high pain group (*n* = 12).

### 2.2. Data and Study Variables

Data were collected for all participants at baseline and after three months, including fasting blood plasma samples, anthropometric measurements for weight, height, waist and hip circumferences, as well as dual-energy X-ray absorptiometry (Discovery A fan beam; Hologic, Marlborough, MA, USA). Plasma samples were tested at the Loma Linda University Medical Center laboratory to determine fasting blood glucose, HbA1c, and lipid profiles (high-density lipoprotein (HDL), low-density lipoprotein (LDL), total cholesterol, as well as triglycerides). All anthropometric measurements were taken twice for reliability, using Lohman et al.’s standardized techniques [22]. In addition, weight and height were assessed using a balance scale (Detecto, Webb City, MO, USA) and a wall-mounted stadiometer (Holtain, Crymych, UK), respectively.

Blood samples were collected at baseline and three months from each participant into EDTA-treated (lavender-top) tubes between 8 and 10:30 am after a 12-h fast. Blood was centrifuged at 2000× *g* at 15 °C for 15 min and immediately aliquoted into sterile polypropylene tubes for plasma collection. Plasma aliquots were sent to Loma Linda University Medical Center for clinical lab analysis, with the remaining aliquots stored in liquid nitrogen at –80 °C for the metabolomics measurements.

Metabolic profiling was performed as previously described [23]. Untargeted semiquantitative metabolomic analysis was performed on three independent platforms: ultra-high-performance liquid chromatography (HPLC)/tandem mass spectrometry (MS) optimized for basic species, UHPLC/MS/MS optimized for acidic species, and gas chromatography (GC). Metabolites were identified by comparing the ion features in the experimental samples to a reference library of chemical standards that included retention time, molecular weight (*m*/*z*), preferred adducts, and in-source fragments, as well as associated MS. In addition, biochemical features were curated by visual inspection for quality control using the software developed at Metabolon [24].

To ensure that changes detected in the untargeted metabolomic analysis were secondary to the dietary intervention, we monitored participants’ diet, medication, and exercise habits throughout the three-month intervention. Furthermore, we implemented a paired analysis statistical approach measuring changes from individuals’ baseline values [10], which control for extraneous sources of variation. Untargeted metabolomics allows for the detection of very subtle alterations in biochemical pathways [25].

### 2.3. Statistical Analysis

The *p* values reported are unadjusted for false discovery rate (*q*-values). However, in an analysis of 695 biochemicals with an assumption that most would fulfill the null hypothesis, approximately 30 would be expected to achieve a *p*-value less than 0.05 by chance due to multiple hypothesis testing. *Q* values consider false discovery rate and increase with the number of statistical tests performed. Rather than sorting by *p*-value and redefining a threshold below 0.05 to achieve more stringent statistical confidence, we have included all biochemicals that reached a *p*-value less than 0.05 in our subsequent pathway analyses because a higher *q* value does not necessarily exclude the significance of a result. We further analyzed the metabolomic data using random forest (RF), a supervised classification technique based on an ensemble of decision trees [26]. This machine-learning classification method was performed using sklearn (version 0.24.1) in Python (version 3.8.8).

We also performed a hierarchical clustering analysis (HCA) to understand how our intervention leads to grouping the data and assessed differences amongst the groups (baseline and three months) where individuals served as their own controls. Specifically, we evaluated complete clustering using the Euclidean distance, in which each sample is a vector with all the metabolite values. Thus, the differences seen in the cluster may be unrelated to the treatment groups or study design.

An occupational threshold of 70% was applied to metabolomics data, requiring biochemicals present in at least 70% of the participants to be considered for analysis. Statistical analyses were performed using SPSS version 28 (IBM, SPSS, Inc., Armonk, NY, USA), Prism 9 (GraphPad Software, San Diego, CA, USA), QIAGEN IPA (QIAGEN Inc., https://digitalinsights.qiagen.com/IPA) (accessed on 11 November 2019)), and MetaboAnalyst 5.0 [27]. Paired-sample *t*-test assessed all other data for normally distributed continuous variables and Wilcoxon signed-rank tests for abnormally distributed continuous variables to determine statistically significant differences from baseline vs. three months post-supplementation. Kolmogorov–Smirnov, and Shapiro–Wilk normality tests, together with the Grubbs’ test, also known as extreme “studentized” deviate (www.graphpad.com accessed on 9 December 2021), were used to investigate outliers and spread. Data are presented as mean ± SD. Statistical differences were considered significant at α = 0.05 unless otherwise specified.

Partial Spearman correlation was performed on top metabolites identified by RF and between patients reporting neuropathic pain. It included SF-MPQ-sensory score and metabolites adjusted for id, including all data points (baseline, and three months). The correlation with SF-MPQ sensory score was based on complete data, excluding missing values.

## 3. Results

### 3.1. Clinical Data of “En Balance-Plus” Participants Pre/Post-Omega-3 PUFAs Supplementation

Participant characteristics and clinical data were collected at baseline and after three months of DHA-enriched fish oil supplementation (Table 1). The intervention was associated with a small but significant decrease in hemoglobin A1c (HbA1c) percentage. HbA1c percent decreased from 7.6 to 7.4 (*p* = 0.014). Body mass index (BMI), LDL, HDL, fasting blood glucose, and total cholesterol were unchanged during the intervention. Participants also completed food frequency questionnaires (FFQ), which allowed for estimating dietary omega-3 fatty acids consumption at baseline and three months (Table 2). The self-reported data showed that while the participants had low plasma DHA and EPA dietary intake at baseline, there was a significant increase in consumption after three months (*p* = 0.0001). The FFQ data were further supported with the untargeted metabolomics finding that showed a significant increase in the levels of DHA and EPA in the plasma following the three months of the study (Table 2, *p* ≤ 0.001).

In a previous study, we found that participants reporting neuropathic pain symptoms, as assessed by the SF-MPQ, had a significant reduction in SF-MPQ sensory and affective scores at the final evaluation after three months of omega-3 PUFA supplementation (change from baseline −5.3 (*p* < 0.001) and −1.3 (*p* = 0.012), respectively) [10]. Additionally, the data showed that increased plasma DHA levels significantly correlated with improved SF-MPQ sensory scores (r = 0.425, *p* = 0.030) [10].

### 3.2. Metabolomics Data Analysis

Untargeted metabolomics analysis identified 695 biochemicals of known identity. However, only a total of 106 met the occupational threshold of 70%. In addition, the levels of 69 transformed biochemicals were significantly increased (*p* < 0.05) at three months on the matched pairs *t*-test, while 37 were significantly decreased (*p* < 0.05) (Table 3). Significant metabolites changes are represented by percent change ((V_2_ − V_1_)/|V_1_|) × 100.

**Figure 1:**
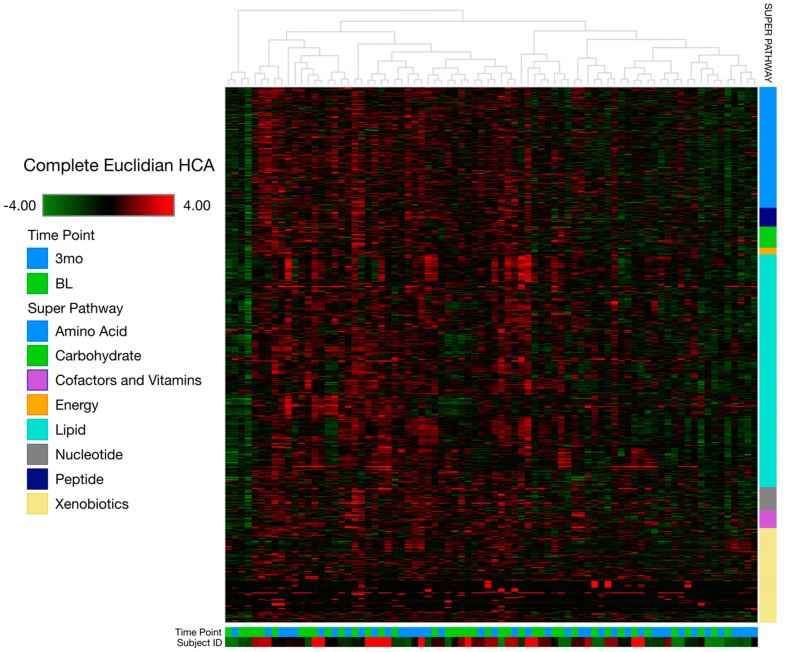
Dendrogram from Hierarchical Cluster Analysis. Baseline and three-month samples from the same subject clustered together in multiple instances. Samples from the same time point had a moderate tendency toward adjacency.

Outcomes associated with this type of intervention may be influenced by the identity of the subject and changes that occurred within each volunteer from baseline to three months. We used (HCA) to assess the relative magnitude of these two factors. As displayed in Figure 1, the dendrogram showed a mixture of clusters from the same time point and the same subject. Additionally, it appears that before and after samples from the same subject cluster together in many instances, suggesting that the intervention did not cause sweeping metabolic changes within a given individual.

**Figure 2:**
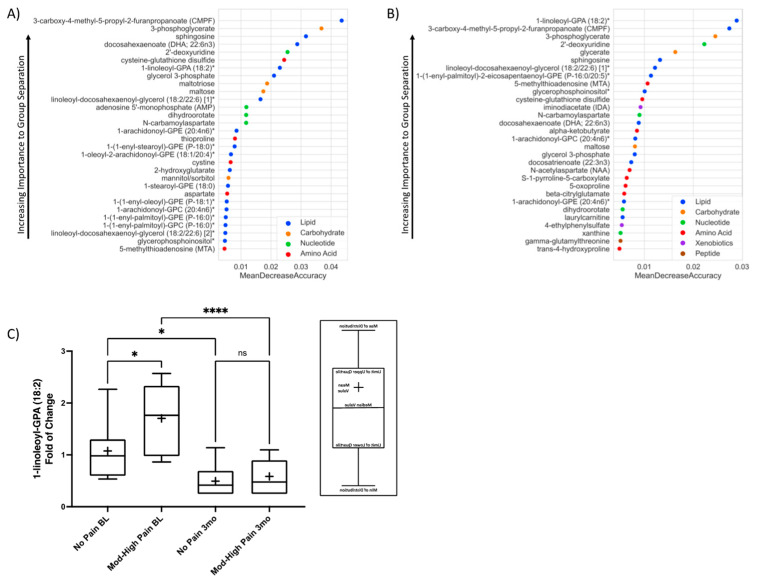
RF classification of plasma samples collected at baseline and 3-months after omega-3 PUFA supplementation. (**A**) Classification of all participants’, regardless of pain status, metabolites were 93% accurate for samples. Top factors contributing to group separation shown in the biochemical importance plot. (**B**) Classification of all participants’, who reported pain symptoms, metabolites were 92% accurate for samples. (**C**) A one-way ANOVA revealed that there was a statistically significant difference in mean 1-linoleoyl-GPA (18:2) relative plasma levels (fold change) between at least two groups (F(3, 42) = (14.63), *p* ≤ 0.0001). Bonferroni’s post hoc analyses showed significant 1-linoleoyl-GPA (18:2) metabolite plasma level change when comparing No Pain BL to Mod-High Pain BL groups (*p* = 0.0143, 95% C.I. = (−1.158, −0.097)), No Pain BL to No Pain 3mo groups (*p* = 0.0220, 95% C.I. = (0.063, 1.100)), and Mod-High Pain BL to Mod-High Pain 3mo (*p* ≤ 0.0001, 95% C.I. = [0.578, 1.66]). * *p* ≤ 0.05 and **** *p* ≤ 0.0001.

Next, to understand feature importance attributed to the omega-3 intervention, we performed random forest classification using metabolite values as predictors for classifying samples at baseline or post-intervention. Classification of plasma samples collected at baseline and following fish oil intervention was 93% accurate in sorting samples when a value of 50% would be expected by random chance. Metabolite importance for group classification was expressed as a mean decrease in accuracy, plotted in Figure 2A. The top 30 metabolites contributing to group separation were mainly enriched with lipid compounds followed by amino acids, nucleotides, and carbohydrate biochemicals.

As we included participants reporting pDN symptoms and no pain symptoms, we also analyzed the metabolome of the pain group at baseline and post-intervention, Figure 2B. We did not find major differences among top features post RF analysis. The findings in the pain RF shared over 50% of the top metabolites that were also expressed in the overall RF analysis, which included all participants, regardless of pain status. However, RF identified 1-linoleoyl-GPA (18:2) as an important metabolite specifically in the pain RF analysis. To understand the significance of 1-linoleoyl-GPA (18:2) as the top feature in the pain group, we compared relative plasma levels between the pain and no pain groups at baseline. There was no significant difference in relative 1-linoleoyl-GPA (18:2) plasma levels (fold change) between pain baseline group (*N* = 26 M = 1.492, SD = 0.6763) and no pain group (*N* = 12, M = 1.076, SD = 0.581); t(24.78) = 1.946, *p* = 0.614). Considering that the pain group is not homogenous, as described in our previous publication [10], we tested 1-linoleoyl-GPA (18:2) plasma levels between the mod-high pain vs. no pain groups. The relative plasma level of 1-linoleoyl-GPA (18:2) was significantly different between the mod-high pain vs. no pain groups at baseline and three months, Figure 2C.

After identifying principal metabolites associated with the intervention, we characterized the major metabolic pathways involved with our omega-3 supplementation. The significant pathways involved cellular oxidation, neurotoxicity, phospholipid, and acylcarnitine metabolism. We describe the metabolic maps below.

#### 3.2.1. Omega 3 PUFAs Effect on Metabolites Associated with the Overall Cellular Oxidative State

**Figure 3:**
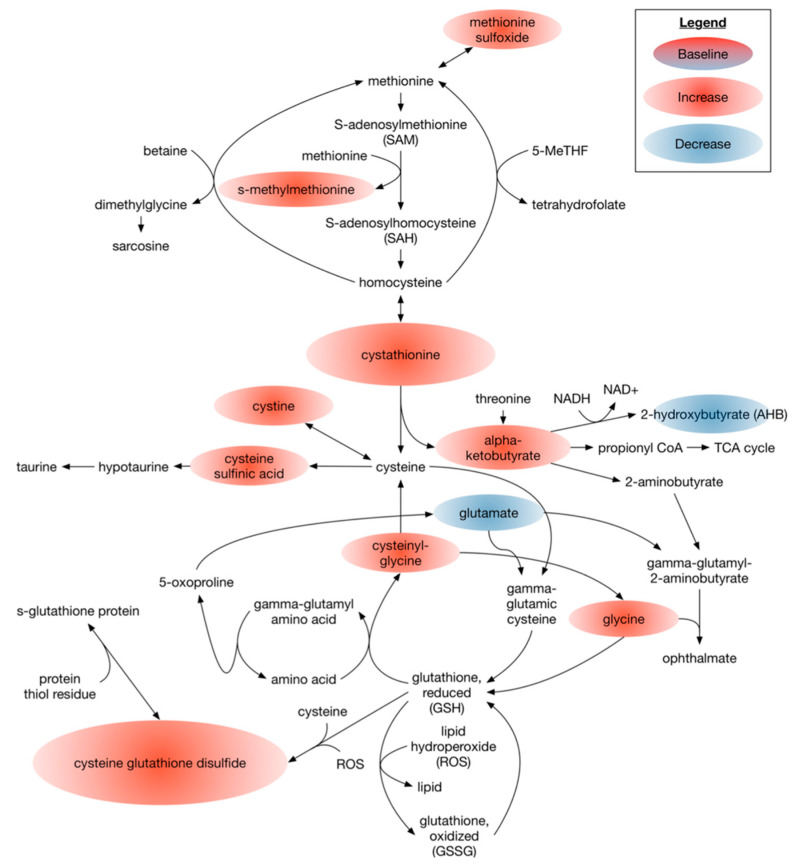
Changes in cysteine, methionine, and glutathione metabolism overlaid on a metabolic map. The size of each colored bubble is proportional to the fold change (increase, red; decrease, blue). The baseline bubble in the legend corresponds to a fold change of 1. 5-MeTHF, 5-Methyltetrahydrofuran-2-ol; NADH, nicotinamide adenine dinucleotide (NAD) + hydrogen (H); NAD, nicotinamide adenine dinucleotide; Propionyl CoA, propionyl coenzyme A; TCA cycle, tricarboxylic acid cycle.

Increased reactive oxygen species (ROS) and decreased antioxidant defenses have been implicated in the pathogenesis of diabetic neuropathy [28,29,30]. Remarkably, glutathione concentration is reduced in patients with type 2 diabetes [31,32], exposing the sensory pain axis to biochemicals promoting neurodegeneration. Notably, our dietary intervention significantly increased several metabolites involved in the production of glutathione. Specifically, we found a dramatic increase in the relative plasma level of cystathionine (+90%), a precursor of cysteine (Table 4). Additionally, s-methylmethionine (+7%) and glycine (+11%) significantly increased after the dietary supplementation. Interestingly, indicators of oxidative stress, cysteine-glutathione disulfide (+156%) and cysteinylglycine (+19%), were increased at the three-month time point. However, after dietary intervention, 2-hydroxybutyrate, previously known to be increased in acute oxidative stress pathologies, significantly decreased by 18% [33]. Thus, it appears that the methionine-cysteine-glutathione axis functions to modulate the chronic oxidative stress given the decrease in 2-hydroxybutyrate production. Both cysteine and methionine metabolism supported glutathione production (Figure 3). These findings align with our previous report on dietary omega-3 PUFAs augmenting glutathione turnover in our spinal cord injury model [14].

#### 3.2.2. Omega-3 PUFAs Effects on Biomarkers for Neurotoxicity

It is well known that neuronal excitotoxicity is associated with increased oxidative stress and neuronal apoptosis [34]. This cellular process is at least partly mediated by sustained activation of the NMDA receptor [35]. A key neurotransmitter associated with excitotoxicity is glutamate [36]. Activation of peripheral nervous system glutamate receptors contributes to mechanical hyperalgesia in neuropathic pain animal models [36,37]. We found that plasma glutamate levels (−11%) decreased post dietary intervention. In contrast, the metabolomic data demonstrated a significant increase of glycine (+11%) and arginine (+10%) in the plasma suggesting that omega-3 PUFAs trigger a rebalancing of neurotoxic amino acids (Table 4), metabolic map not shown.

#### 3.2.3. Dietary Omega-3 Supplementation Regulates Phospholipid Profiles in Plasma of Patients with Type 2 Diabetes

**Figure 4:**
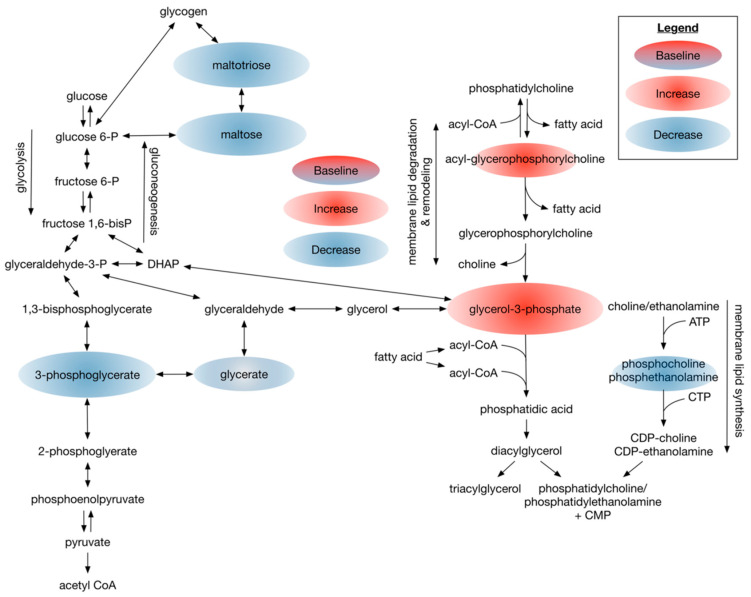
Glycerolipid and glucose metabolism changes overlaid on a metabolic map. The size of each colored bubble is proportional to the fold change (increase, red; decrease, blue). The baseline bubble in the legend corresponds to a fold change of 1. glucose-6-P, glucose-6-phosphate; fructose-6-P, Fructose-6-phosphate; fructose 1,6 bisP, fructose 1,6, bisphosphate; glyceraldehyde-3-P, glyceraldehyde-3-phosphate; DHAP, Dihydroxyacetone phosphate; acyl-CoA, acetyl coenzyme A; ATP, adenosine triphosphate; CTP, Cytidine triphosphate; CDP-choline, cytidine diphosphate-choline; CDP-ethanolamine, cytidine diphosphate-ethanolamine; CMP, cytidine monophosphate.

We further analyzed the biochemicals involved in lipid membrane homeostasis. A hallmark of certain patients with type 2 diabetes can be an elevated plasma level of saturated (palmitic acid) free fatty acids. With this elevation, the flexibility of membranes decreases, and multiple functions associated with electrical conduction, as well as signal transduction, are compromised [38]. Due to this, we were interested in the composition shift of diacylglycerols post the DHA-enriched dietary intervention. As expected, phospholipids associated with improved membrane fluidity were increased (Table 5). Specifically, a precursor for phospholipid synthesis, linoleoyl-docosahexaenoyl-glycerol (18:2/22:6) plasma level (+252%), significantly increased. Additionally, the dietary intervention significantly decreased 3-phosphoglycerate (3PG) and glycerate levels, −57% and −15%, respectively (Figure 4). These findings, coupled with increases in glycerol-3-phosphate (+78%), diacylglycerols, and glycerophospholipids (e.g., linolenoyl-GPC), suggests that excess glucose and free fatty acids were diverted to synthesize phospholipids (Figure 4). Lastly, as predicted, the n6 fatty acid arachidonic acid plasma level decreased by 17%, and phospholipids containing arachidonate also significantly decreased post dietary intervention (see Figure 4 and Table 5).

#### 3.2.4. Dietary DHA-Enriched Supplementation Increases Acylcarnitine Species in Participants Plasma

**Figure 5:**
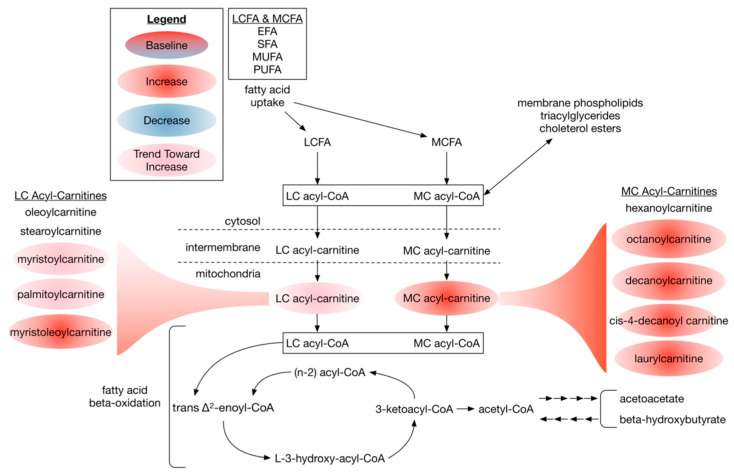
Changes in oxidation of fatty acids overlaid on a metabolic map. The size of each colored bubble is proportional to the fold change (increase, red; decrease, blue). The baseline bubble in the legend corresponds to a fold change of 1. C Acyl-Carnitines, Long Chain Acetyl-Carnitine; LCFA, Long Chain Fatty Acids; MCFA, Medium Chain Fatty Acids; EFA, Essential Fatty Acids; MUFA, Monounsaturated Fatty Acids; PUFA, Polyunsaturated Fatty Acids; LC acyl-CoA, Long Chain acetyl-coenzyme A; MC acyl-CoA, Medium Chain acetyl-coenzyme A; MC acyl-carnitine, Medium Chain acetyl-carnitine.

A predictive metabolic outcome of an omega-3 rich dietary supplementation would include biochemicals involved in fatty acid oxidation. Carnitine derivatives have been demonstrated to play a pivotal role in neuroregeneration [39]. Specifically, L-carnitine and its products, acetylcarnitine (ALC) and propionyl-carnitine (PLC), are shown to have beneficial effects on neuropathic pain, degenerative axonal changes, as well as nerve fiber regeneration in clinical studies [40]. In our dietary intervention, we found a significant increase in several medium- and long-chain acylcarnitine species, including octanoyl carnitine (+39%), decanoylcarnitine (+37%), cis-4-decenoyl carnitine (+21%), laurylcarnitine (31%), as well as myristoleoylcarnitine (+27%), see Figure 5. Interestingly, AMP, a marker of energy stress, was significantly lower following the dietary intervention (Table 6).

### 3.3. Ingenuity Pathway Analysis (IPA)

We analyzed all significantly different metabolites, which were present in at least 70% of the participants, using QIAGEN IPA (QIAGEN Inc., https://digitalinsights.qiagen.com/IPA (accessed on 11 November 2019)) to clarify the biological significance of the experimental data [41]. Of the 106 metabolites significantly different from baseline, only 90 were recognized by the database. We obtained information regarding related bio-functions from these metabolites. The results of the IPA analysis showed significant involvement in several bio-functions, such as free radical scavenging, cellular function and maintenance, cell signaling, as well as molecular transport. We categorized the top nine enriched bio functions (*p* < 5.0 × 10^−4^ and z-score >1.5 or <−1.5) in Table 7. Interestingly, formation of reactive oxygen species (*p* = 4.38 × 10^−4^, z-score = −1.96), peroxidation of lipids (*p* = 2.24 × 10^−5^, z-score = −1.944), entrance of Ca^2+^ (*p* = 1.55 × 10^−4^, z-score = −1.969), and excitation of neurons (*p* = 1.07 × 10^−4^, z-score = −1.091) are all suggested to be downregulated. In contrast, metabolites associated with the concentration of glutathione (*p* = 3.06 × 10^−4^, z-score = 1.974) in cells significantly increased.

### 3.4. The Associations between Top Factors Contributing to Group Separation Per RF and SF-MPQ Sensory Score

Although IPA can define essential pathways associated with our intervention, these identified pathways may not be directly associated with improved pain symptoms. Because of this, we compared top factors identified by the RF analysis to participants reporting neuropathic pain symptoms, as measured by the SF-MPQ. Several metabolites correlated with SF-MPQ sensory scores, Table 8. As expected, sphingosine and 1-linoleoyl-GPA (18:2) positively correlated with sensory scores, signifying that higher levels of both sphingosine and 1-linoleoyl-GPA (18:2) are associated with severe pain symptoms. Interestingly, both metabolites significantly decreased post-intervention, Table 9. These findings are of particular significance since both sphingosine and 1-linoleoyl-GPA (18:2) are implicated in the pathogenesis of neuropathic pain [42,43]. Furthermore, metabolite cysteine-glutathione disulfide was inversely correlated with SF-MPQ sensory score.

## 4. Discussion

Omega-3 PUFAs have anti-inflammatory and antioxidant properties, and supplementation has been shown to effectively ameliorate traumatic and chronic conditions such as spinal cord injury, traumatic brain injury, and now painful neuropathy in T2DM [15,40,44,45]. Both pre-existing, pre-clinical, and emerging clinical data raise the potential of using omega-3 PUFAS as a primary or complementary treatment of several human chronic and traumatic conditions. The lack of toxicity and the ready availability of omega-3 PUFAs suggest an exciting avenue for therapy conditions associated with both acute and chronic pain [11,15,46,47,48]. To date, most studies with omega-3 PUFAs have been largely descriptive. Our present study defines underlying omega-3 PUFA targets through unbiased interrogation of the plasma metabolome. We report that omega-3 PUFA supplementation profoundly improves participants’ plasma neurorestorative and antioxidant metabolomic profiles. This improvement was evidenced by marked and selective changes in the metabolites associated with glycerolipid, omega-3 PUFA, glucose, and cysteine/methionine/glutathione metabolism.

The pathophysiology of pDN involves a series of complex cascades that are interrelated and ultimately result in neural dysfunction [49]. Several of these processes include inflammation, cell death and dysfunction, altered peripheral blood flow, impaired spinal inhibitory function, neurotransmitter, ionic imbalances, compromised energy metabolism, as well as production of free radicals [50]. Thus, our central hypothesis is that therapeutic interventions mitigating several pro-neuropathic pain processes may be required to alleviate neuropathic pain.

### 4.1. Overall Metabolome Indicates Targeted Metabolomic Changes

Sensitive characterization of metabolic changes associated with targeted dietary interventions is made possible with current metabolomics technology. Within the field of metabolomics, significant interest has been given to T2DM because of its extensive metabolic disturbances [51,52,53,54,55,56,57,58]. Therefore, we applied GC-MS-based metabolomics analysis to understand changes occurring during an omega-3 PUFA dietary intervention. We compared metabolite profiles of a Hispanic type 2 diabetes cohort with a reported low nutritional intake of omega-3 fatty acids [10] at baseline and after three months to identify changes in clinical outcomes, key metabolites, and metabolic pathways. This report is the first to use untargeted metabolic profiling to characterize changes in plasma metabolites associated with omega-3 fatty acid supplementation in a population known to have low omega-3 fatty acid intake.

Considering the limitation of the one-arm design of our intervention, we sought to determine whether differences were attributable to the oral supplementation. The dendrogram from hierarchical cluster analysis (Figure 1) showed a moderate tendency for samples from the same time point to cluster together (baseline and three months). However, samples from the same subject taken before and after supplementation also clustered together in many instances. This clustering indicates that a subset of critical metabolic pathways contributed to group differences rather than sweeping metabolic changes.

We used Random Forest ensemble learning, a robust statistical technique for accurately classifying subjects from relatively small learning datasets, to understand the most critical metabolic features for group separation between time points [59]. In Figure 2A, the Random Forest classification of plasma samples, according to timepoint, was highly accurate in classification (93%). It showed the importance of DHA, related glycerolipids, and carbohydrate metabolites in reliably differentiating between samples from before and after the intervention. These results suggest that the dietary intervention altered targeted metabolic pathways. Furthermore, the top features important for group separation were similar between the pain group and the overall sample pool, as demonstrated by RF. This suggests the overall pathways were similar, save for metabolites such as 1-linoleoyl-GPA (18:2).

Moreover, dietary DHA-enriched supplementation, as expected, did not markedly change clinical values and anthropometric measurements. Notably, fasting blood glucose, LDL, BMI, cholesterol, HDL, triglycerides, and cholesterol: HDL ratio did not significantly change. There was a small effect size on HbA1c, but the time interval and small degree of change would not be predictive of clinical benefits. These data, taken together with no reported dietary or medication modifications by participants, except for the provided DHA-enriched supplementation, suggest that changes observed in the metabolic profile can be attributed to the study’s dietary intervention.

### 4.2. Dietary DHA-Enriched Supplementation Leads to Improved Antioxidant Metabolic Plasma Profiles of Participants with Type 2 Diabetes

Although the measurement of plasma metabolites does not directly assess cellular metabolism, plasma metabolites are highly sensitive to changes in cellular metabolism and models of overflow metabolism support the close relationship between intracellular and extracellular metabolic states [60,61]. Additionally, others have demonstrated that untargeted metabolomics of plasma samples can generate significant insights regarding health status assessment and disease management [62].

While generally considered a sign of increased oxidative stress, as indicated by elevated cysteine glutathione disulfide levels in our subjects, glutathionylation may be a preferable, protective alternative to irreversible oxidation of intracellular proteins [63,64,65,66]. Interestingly, 2-hydroxybutyrate, a well-established marker of oxidative stress [67,68,69], was decreased in our cohort following fish oil supplementation. Moreover, while indicators of reversible oxidative stress, such as cysteine-glutathione disulfide and cysteinylglycine, were increased post-intervention, omega-3 PUFA supplementation was associated with a significant reduction of 2-hydroxybutyrate (Figure 5). Thus, the increased omega-3 PUFAs might have driven the increase in the methionine-cysteine-glutathione axis to address the pro-oxidative stress phenotype found at baseline [55]. Furthermore, IPA demonstrated downregulation of reactive oxygen species production and lipid peroxidation while increasing the glutathione system’s activity. Together, these findings suggest that the participants’ pro-oxidative stress profile at baseline dramatically changed to a more antioxidant phenotype.

Oxidative stress is a signature of type 2 diabetes and a significant player in the pathogenesis of diabetic neuropathy. Lipotoxic stress and hyperglycemia auto-oxidation initiate extensive generation of reactive oxygen species, and lipid peroxidation overwhelming the nerve, dorsal root, as well as sympathetic ganglia antioxidant capacity [29,30,70,71,72]. Ultimately, increased oxidative stress leads to widespread nerve-cell dysfunction. Our previous work demonstrated that dietary DHA-enriched supplementation significantly improved volunteers’ neuropathic pain scores [10]. This follow-up global metabolomic analysis shows that a dietary supplementation enriched with omega-3 PUFAs can dramatically increase the antioxidant capacity in patients with type 2 diabetes.

### 4.3. Dietary DHA-Enriched Supplementation Modulates Circulating Excitotoxic Amino Acids

NMDA receptors play an important role in central sensitization in neuropathic pain [73]. Particularly, persistent over-stimulation of NMDA-type glutamate receptors leads to excitotoxic neuronal death and is essential for the long-term plastic changes in the spinal dorsal horn and the development of diabetic neuropathic pain [74,75,76]. Preliminary evidence in animal models has shown that omega-3 fatty acids may inhibit NMDA receptor activity in the context of inflammatory pain states [77]. Interestingly, glutamate levels in the plasma increase in patients with type 2 diabetes and are elevated in chronic neuropathic pain conditions [78,79]. Thus, our dietary intervention seems to have modulated the glutamatergic systems to a more neuroprotective state by decreasing glutamate levels. Furthermore, given the established role of GABAergic and glycinergic dysfunction in the development of neuropathic pain [80,81,82], increased levels of plasma glycine and arginine support the indication that our DHA-rich supplementation improves neurorestorative pathways [83,84,85]. Moreover, taking what was found in the IPA, which indicated improved calcium recycling and decreased excitation of neurons (data not shown), points to the neuroprotective properties of our DHA-enriched supplementation in our type II diabetes cohort.

### 4.4. Dietary DHA-Enriched Supplementation Changes Phospholipid Composition and Increases Acyl-Carnitine Levels

In type II diabetes, increased free fatty acids lead to high cytoplasmic saturated fatty acyl-CoA, which allosterically inhibits fatty acid desaturases and reduces the synthesis of PUFA [86]. In our Mexican American population, we have previously reported dramatic low levels of dietary omega-3 and elevated levels of saturated fats [87]. In the context of neuropathic pain, this nutritional deficit can lead to neuronal dysregulation. Specifically, when cellular membranes are overly composed of saturated fatty acyl-CoA, the flexibility decreases and multiple functions associated with electrical conduction and signal transduction may become dysregulated [38]. Our untargeted metabolomic analysis found a dramatic shift in phospholipid metabolism, suggesting increased PUFA incorporation into cellular membranes. Interestingly, palmitate levels did not change during the intervention. Therefore, it appears that merely increasing the levels of omega-3 PUFAs can restore membrane homeostasis without altering levels of saturated fatty acids.

In type II diabetes, there is typically an oversupply of lipids resulting in the accumulation of bioactive lipid metabolites. It has been proposed that Schwann cells (SC) may shift the stoichiometry of their lipid production in pDN, increasing toxic ceramide production [71]. This proposed overproduction of ceramide is mediated by diabetic SC undergoing loss of oxidative phosphorylation and ATP production due to excess acyl-CoAs undergoing metabolic reprogramming, leading to an accumulation of acylcarnitines [71]. SC transport of these acylcarnitines is toxic to dorsal root ganglion neurons [88], accounting for the development of pDN [71]. Interestingly, our untargeted metabolomics analysis showed an increase of medium and long-acylcarnitines, but with associated decreases in lipid peroxidation and formation of reactive oxygen species. Furthermore, we observed a significant reduction in plasma sphingosine levels, a precursor of ceramide synthesis, suggesting a decrease in ceramide production. DHA treatment in a neuronal culture model has been previously reported to inhibit palmitic acid-induced mitochondrial membrane depolarization altogether [13]. Dietary omega-3 supplementation appears to correct the proposed metabolic reprogramming associated with pDN, leading to increased phospholipid biosynthesis and improved mitochondrial bioenergetics, supported by a decreased AMP.

Lastly, our previous studies showed that dietary omega-3 PUFAs dietary supplementation attenuated the development of thermal hyperalgesia following SCI and improved neuropathic pain scores in participants with type II diabetes [7,10]. In agreement with data presented in this study, our previous metabolomic profiling and IPA identification of metabolite biological function of SCI tissue demonstrated a robust increase in glutathione concentration, improved calcium regulation, and decreased neuronal excitation [14]. Together, these untargeted metabolomics data sets, as well as animal and human bio-samples, are highly suggestive that dietary DHA supplementation can directly impact neurorestorative and antioxidant pathways in chronic inflammatory disease states.

### 4.5. Top Feature Importance Metabolites Correlate with SF-MPQ Sensory Score

We previously demonstrated that DHA relative plasma concentration change correlated with improved SF-MPQ sensory score [10]. Additionally, we now report that principal metabolites identified by RF analysis correlate with improved SF-MPQ sensory scores. Sphingosine and 1-linoleoyl-GPA (18:2) correlated with higher SF-MPQ sensory scores. These two findings are significant. First, 1-linoleoyl-GPA (18:2) and it’s signaling have been demonstrated to play a pivotal role in the pathogenesis of neuropathic pain [42,89]. We found that linoleoyl-GPA (18:2) was significantly higher in our mod-high pain group and significantly decreased after the dietary intervention. Second, we previously reported that sphingosine significantly decreased in the pain group [10]. Similarly, sphingolipid metabolism dysregulation is associated with pDN [90]. These findings support that the SF-MPQ captured participants with neuropathic pain, and our omega-3 dietary intervention reversed known metabolic markers for pDN.

### 4.6. Potential Uses of Omega-3 Intervention beyond Pain

Interestingly, Latinos appear to have low omega-3 PUFA intake within their diets and they suffer from other increased microvascular complications including nephropathy and retinopathy [87,91,92]. Our supplementation study suggests that this “nutritional deficit” may promote increasing incidence of microvascular complications and that a nutritional approach to increasing omega-3 PUFA intake could be a non-medication approach to decrease adverse outcomes.

Another significant comorbidity of type 2 diabetes is increased susceptibility and poor outcomes to respiratory viral infections [93,94] such as influenza. Of current concern, patients with type II diabetes infected with SARS-CoV-2 and who develop COVID-19 have a worse prognosis than nondiabetics [95,96]. Evidence suggests that an at-risk population may benefit from an anti-inflammatory diet [97]. One component of an anti-inflammatory diet includes omega-3 fatty acids. Given our current findings that our DHA-rich supplementation improved the antioxidant metabolomic profile of our participants and the known inflammatory storm caused by COVID-19 [96], adding omega-3 PUFAs to patients’ diets with type 2 diabetes may serve to mitigate the effects of COVID-19.

### 4.7. Study Limitations and Strengths

We have previously discussed limitations to this study [10]. Briefly, we recognize that this analysis lacks a traditional control group. Volunteers participated in an interactive and supportive environment, potentially introducing the Hawthorne or placebo effect regarding reported SF-MPQ pain scores. As our method of collecting neuropathic pain symptoms were based on the SF-MPQ, a definitive diagnosis of pDN was not established, nor were all other potential causes of peripheral neuropathy ruled out. Further, we cannot completely rule out selection bias, which affects this cohort of participants regarding both eligibility and selection criteria.

The above confounders complicate outcome analysis of many community-based interventions due to the lack of a reliable quantitative readout of given intervention and when at times the selection of a control group may comprise the overall objective of the intervention [98]. We diminished many of these extraneous sources of variation and account for the effect of participating in our diabetes-intervention program by assigning participants as their own controls and measuring changes from their individual baseline values [99]. Another strength is the metabolic readout of the dietary intervention. Our untargeted metabolomics analysis confirms that the omega-3 PUFA supplementation altered several known pathways involved with omega-3 PUFAs metabolism.

Lastly, even though we did not use gold standard diagnostic techniques to diagnose DN, metabolites known to be involved in the pathogenesis of DN were elevated in our pain group compared to participants not reporting pDN symptoms, suggesting that we indeed captured participants with true pDN.

## 5. Conclusions

We demonstrated that dietary DHA-enriched supplementation has a wide-ranging impact on the antioxidant metabolomic profile of our participants. Overall, this study shows that untargeted metabolomics is sensitive for defining targeted alteration in metabolism secondary to a dietary intervention. Specifically, we found that a dietary DHA-enriched supplementation improved metabolic profiles regarding omega-3 PUFA metabolism, glycerolipid metabolism, cysteine, methionine, glutathione metabolism, and glucose and fatty acid homeostasis without changing clinical parameters. Furthermore, circulating plasma metabolites showed a significant shift toward decreased reactive oxygen species biosynthesis, lipid peroxidation, improved Ca^2+^ homeostasis, and increased glutathione activity. These changes may explain previously reported reduction in neuropathic pain symptoms in our En Balance plus cohort [10]. Since omega-3 PUFA supplementation is safe in patients with type 2 diabetes, future studies are warranted to define further its role in the possible improvement of pDN symptoms [100].

## Figures and Tables

**Table 1:** Characteristics of study participants (*n* = 35).

Clinical Parameter	Baseline (BL)	3 Months (3Mo)	Difference 3Mo—BL	Significance, *p*
Age (year)	55.5 ± 11.8	-	-	-
Male%, Female%	43%, 57%	-	-	-
% Hispanic	100	-	-	-
BMI, kg/m^2^	29.7 ± 5.5	29.8 ± 5.7	0.1	0.706 ^1^
Cholesterol, mg/dL	176.3 ± 35.7	179 ± 34.3	2.7	0.294
LDL, mg/dL	117.1 ± 32.5	111.6 ± 27.9	−5.5	0.083
HDL, mg/dL	47.2 ± 13	48.2 ± 14	1	0.600
Cholesterol:HDL	3.9 ± 1.0	3.9 ± 1.1	0	0.561
Triglycerides, mg/dL	169.3 ± 83.9	163.7 ± 123	−5.6	0.347
Fasting Glucose, mg/dL	154.1 ± 71.9	142.7 ± 65.5	−11.4	0.095
HbA1c%	7.6 ± 2.3	7.4 ± 2.1	−0.2	0.014

Percentage of individuals or means ± SD of participants is shown for each variable and each time point. ^1^ Normally distributed. BMI, Body mass index; LDL, low-density lipoprotein; HDL, high-density lipoprotein.

**Table 2:** En Balance-Plus participants reported dietary and GC-MS relative omega-3 PUFAs levels at baseline and three months (*n* = 40).

	Baseline (BL)	3 Months (3Mo)	Difference 3Mo—BL	Significance, *p*
GC-MS				
DHA	0.71 ± 0.4	1.85 ± 1.1	1.114	<0.001
EPA	1.03 ± 0.68	1.62 ± 1.1	0.59	0.001
Dietary Intake				
DHA, mg	57.6 ± 98.9	1035.2 ± 30.1	977.6	0.0001
EPA, mg	26.8 ± 65.5	211.7 ± 11.3	184.9	0.0001

Percentage of individuals or means ± SD of participants is shown for each variable and each time point. GC-MS, Gas chromatography–mass spectrometry; DHA, Docosahexaenoic acid; EPA, Eicosapentaenoic acid.

**Table 3:** Untargeted metabolomic compound identification and statistical comparison.

Total Biochemicals Identified	695
Total Biochemicals *p* ≤ 0.05, matched paired *t*-Test	106
Biochemicals (↑↓)	**69**|**37**

**Table 4:** Metabolites Related to Cellular Oxidative State.

Super Pathway	Sub Pathway	Biochemical Name	Fold Change 3Mo BL	% Change
Amino Acid	Glycine, Serine, and Threonine	glycine	1.11	+11%
	Glutamate	glutamate	0.89	−11%
		glutamine	1.07	-
	Leucine, Isoleucine, and Valine	isoleucine	1.09	-
	Methionine, Cysteine, SAM, and Taurine	methionine	1.1	-
		s-methylmethionine	1.07	+7%
		methionine sulfoxide	1.19	+19%
		cystathionine	1.9	+90%
		alpha-ketobutyrate	1.36	+36%
		2-aminobutyrate	0.94	-
		cysteine	1.01	-
		cystine	1.2	+20%
		cysteine s-sulfate	0.86	−14%
		hypotaurine	0.94	-
		taurine	0.93	-
		2-hydroxybutyrate/2-hydroxyisobutyrate	0.82	−18%
	Arginine and Proline	Arginine	1.1	+10%
	Glutathione	cysteine-glutathione disulfide	2.56	+156%
		cysteinylglycine	1.19	+19%
Carbohydrate	Fructose, Mannose, and Galactose	fructose	0.8	-
Lipid	Glycerlipid	glycerol 3-phosphate	1.78	+78%

Significant metabolite increases and decreases are highlighted in red and blue, respectively.

**Table 5:** Metabolites of glucose and glycerolipid metabolism.

Super Pathway	Sub Pathway	Biochemical Name	Fold Change 3Mo BL	% Change
Carbohydrate	Glycolysis, Gluconeogenesis, and Pyruvate Metabolism	glucose	0.92	-
		3-phosphoglycerate	0.43	−57%
		pyruvate	1.09	-
		lactate	0.95	-
		glycerate	0.85	−15%
	Glycogen Metabolism	maltotriose	0.44	−56%
		maltose	0.52	−48%
Lipid	Polyunsaturated Fatty Acid (n3 and n6)	eicosapentaenoate (EPA; 20:5n3)	1.57	+57%
		docosapentaenate (n3 DPA; 22:5n3)	0.97	-
		docosahexaenoate (DHA; 22:6n3)	2.62	+162%
		docosatrienoate (22:3n3)	0.67	−33%
		arachidonate (20:4n6)	0.83	−17%
		adrenate (22:4n6)	0.82	-
	Phospholipid Metabolism	phosphoethanolamine	0.81	−19%
		glycerophosphoinositol	0.5	−50%
		1,2-dipalmitoyl-GPC (16:0/16:0)	1.15	+15%
		1-palmitoyl-2-oleoyl-GPC (16:0/18:1)	1.14	+14%
		1-stearoyl-2-oleoyl-GPC (18:0/18:1)	1.26	+26%
		1-stearoyl-2-linoleoyl-GPC (18:0/18:2)	1.13	+13%
		1-linoleoyl-2-linolenoyl-GPC (18:2/18:3)	1.32	+32%
		1-palmitoyl-2-linoleoyl-GPI (18:2/18:3)	1.22	+22%
		1-oleoyl-2-linoleoyl-GPI (18:1/18:2)	1.42	+42%
		1-stearoyl-2-linoleoyl-GPI (18:0/18:2)	1.18	+18%
		1-stearoyl-2-arachidonoyl-GPE (18:0/20:4)	0.86	−14%
		1-palmitoyl-2-arachidonoyl-GPE (16:0/20:4)	0.86	−14%
		1-palmitoyl-2-stearoyl-GPC (16:0/18:0)	1.15	+15%
		1-palmitoyl-2-oleoyl-GPI (16:0/18:1)	1.23	+23%
		1-oleoyl-2-arachidonyl-GPE (18:1/20:4)	0.7	−30%
	Plasmalogen	1-(1-enyl-palmitoyl)-2-eicosapentaenoyl-GPE (*p*-16:0/20:5)	1.88	+88%
		1-(1-enyl-palmitoyl)-2-palmitoleoyl-GPC (*p*-16:0/16:1)	1.13	+13%
	Glycerolipid	glycerol 3-phosphate	1.78	+78%
		glycerophosphoglycerol	0.56	−44%
	Diacylglycerol	linoleoyl-docosahexaenoyl-glycerol (18:2/22:6) [1]	3.52	+252%
		linoleoyl-docosahexaenoyl-glycerol (18:2/22:6) [2]	4.61	+361%

Significant metabolite increases and decreases are highlighted in red and blue, respectively.

**Table 6:** Metabolites of fatty acid oxidation.

Super Pathway	Sub Pathway	Biochemical Name	Fold Change 3Mo BL	% Change
Lipid	Fatty Acid Metabolism (Acyl Carnitine)	octanoylcarnitine	1.39	39%
		decanoylcarnitine	1.37	37%
		cis-4-decenoyl carnitine	1.2	20%
		laurylcarnitine	1.31	31%
		myristoylcarnitine	1.17	-
		palmitoylcarnitine	1.13	-
		myristoleoylcarnitine	1.26	26%
	Ketone Bodies	acetoacetate	0.64	-
		3-hydroxybutyrate (BHBA)	0.81	-
	Fatty Acid, Monohydroxy	3-hydroxyhexanoate	1.1	-
		3-hydroxyoctanoate	1.3	-
		3-hydroxydecanoate	1.19	-
		3-hydroxylaurate	1.1	-
Nucleotide	Purine Metabolism, Adenosine Containing	adenosine 5′-monophosphate	0.58	42%

Significant metabolite increases and decreases are highlighted in red and blue, respectively.

**Table 7:** Top bio functions reported by IPA of significantly changed metabolites post omega-3 PUFA supplementation.

Bio Function Categories	Diseases or Functions Annotation	*p*-Value	Activation Z-Score	Number of Biochemicals
Free Radical Scavenging	Synthesis of reactive oxygen species	7.21 × 10^−7^	−1.69	13
	Formation of reactive oxygen species	4.19 × 10^−4^	−1.96	4
	Biosynthesis of hydrogen peroxide	4.95 × 10^−5^	−1.95	5
Cellular Compromise, Lipid Metabolism, Small Molecule Biochemistry	Peroxidation of Lipids	2.25 × 10^−5^	−1.94	6
Cell-to-Cell Signaling and Interaction, Hematological System Development and Function, Inflammatory Response	Aggregation of blood platelets	5.7 × 10^−5^	−1.78	7
Carbohydrate Metabolism, Molecular Transport, Small Molecule Biochemistry	Uptake of D-glucose	1.19 × 10^−4^	−1.72	6
Cell Signaling, Molecular Transport, Vitamin and Mineral Metabolism	Quantity of Ca^2+^	1.52 × 10^−4^	−1.83	9
	Entrance of Ca^2+^	2.15 × 10^−4^	−1.97	4
Drug Metabolism, Molecular Transport, Small Molecule Biochemistry	Concentration of glutathione	3.43 × 10^−4^	1.97	5

Significant Bio Functions, increases and decreases are highlighted in red and blue, respectively.

**Table 8:** Partial spearman correlation between SF-MPQ sensory score and top metabolites contributing to group separation per RF, adjusted for ID.

Metabolite	Correlation	*p*-Value
cysteine-glutathione disulfide	−0.460	<0.001
linoleoyl-docosahexaenoyl-glycerol (18:2/22:6) [1]	−0.266	0.059
Sphingosine	0.489	<0.001
3-phosphoglycerate	0.572	<0.001
Glycerol 3-phosphate	−0.163	0.252
1-linoleoyl-GPA (18:2)	0.406	0.003
2′-deoxyuridine	−0.492	<0.001
Maltose	0.360	0.009

**Table 9:** Metabolites associated with Neuropathic Pain.

Super Pathway	Sub Pathway	Biochemical Name	Fold Change 3Mo BL	% Change
Lipid	Sphingolipid Metabolism	sphingosine	0.45	−55%
	Lysolipid	1-linoleoyl-GPA (18:2)	0.43	−57%

Significant metabolite decreases are highlighted in blue.

## Data Availability

Data supporting reported results can be found in a dataset generated during the study. Additional information available upon request.
